# Clinical Outcomes Associated With In-Hospital Versus Delayed Outpatient Initiation of Sacubitril/Valsartan in Patients With Heart Failure With Reduced Ejection Fraction

**DOI:** 10.7759/cureus.107045

**Published:** 2026-04-14

**Authors:** Helen Oletu, Akinyele Oladimeji, Nneka Muoghalu, Kwasi A Opoku, Azeberoje Osueni, Sunday O Arifayan, Ijeoma A Opara, Emeka Okwuokei, Sylviastella Favour Peteranaba

**Affiliations:** 1 Internal Medicine, North Knoxville Medical Center, Powell, USA; 2 Family Medicine, Obafemi Awolowo University, Ile-Ife, NGA; 3 Public Health, University of Liverpool School of Tropical Medicine, Liverpool, GBR; 4 Internal Medicine, University College Hospital, Ibadan, NGA; 5 Internal Medicine, St. Theresa's Hospital, Nkoranza, GHA; 6 Gastroenterology and Hepatology, Lagos University Teaching Hospital, Lagos, NGA; 7 Health Sciences, University of Ilorin Teaching Hospital, Ilorin, NGA; 8 Medicine, Caribbean Medical University, Willemstad, CUW; 9 Internal Medicine, Hywel Dda University Health Board, Aberystwyth, GBR; 10 Health Sciences and Public Health, University of New Haven, West Haven, USA

**Keywords:** arni, guideline-directed therapy, heart failure, mimic-iv, readmission, sacubitril/valsartan

## Abstract

Background: Heart failure with reduced ejection fraction (HFrEF) remains associated with high rates of hospitalization and early readmission, with approximately 20-25% of patients readmitted within 30 days. Sacubitril/valsartan is a key component of guideline-directed medical therapy, but the optimal timing of initiation during hospitalization versus after discharge remains uncertain.

Objective: This study aimed to evaluate whether initiation of sacubitril/valsartan during hospitalization is associated with improved short-term outcomes compared with delayed outpatient initiation.

Methods: This retrospective cohort study used the MIMIC-IV database. Adult patients hospitalized with HFrEF who received sacubitril/valsartan were included. Patients were classified into in-hospital initiation and delayed outpatient initiation groups based on medication timing. The primary outcome was 30-day hospital readmission, and the secondary outcome was in-hospital mortality. Multivariable logistic regression was used to assess associations after adjustment for age, sex, race, and the Charlson Comorbidity Index.

Results: A total of 4,156 hospitalizations were included. There was no significant association between timing of sacubitril/valsartan initiation and 30-day readmission (aOR=0.91; 95% CI: 0.50-1.67; p=0.764). Higher comorbidity burden was associated with increased readmission risk (aOR=1.04; 95% CI: 1.00-1.07; p=0.035). No significant difference in in-hospital mortality was observed between groups.

Conclusion: Timing of sacubitril/valsartan initiation was not associated with short-term outcomes. Comprehensive management strategies beyond the timing of therapy may be important for improving outcomes in this population.

## Introduction

Heart failure with reduced ejection fraction (HFrEF) is one of the most significant worldwide general illness concerns and has been linked with tremendous morbidity, death, and health services [1]. In spite of the high progress achieved in pharmacological treatment, HFrEF patients still have frequent hospitalizations and a high probability of adverse clinical outcomes [2]. Heart failure hospitalization is regarded as one of the critical events in the course of the disease, which may be a pointer to the clinical instability and deterioration of the cardiac dysfunction. In addition, the post-discharge period is a highly susceptible phase as it is marked by a high probability of rehospitalization and death [3,4]. As a result, maximization of guideline-directed medical therapy (GDMT) in the course of hospitalization has emerged as an important measure to enhance patient outcomes and burden prevention of heart failure [5].

Angiotensin receptor-neprilysin inhibitor (ARNI) therapy can be of value in heart failure patients with HFrEF based on post hoc analysis and subgroup analysis of heart failure studies [6]. Sacubitril/valsartan has been shown to significantly reduce mortality, hospitalization, and rehospitalization of heart failure compared with enalapril in patients with HFrEF [7,8]. Also, the advantages associated with sacubitril/valsartan in the management of patients with HFrEF are evident in the mortality and disease progression reduction, cardiac remodeling, and quality of life improvement [9].

Evidently, sacubitril/valsartan significantly reduced the primary endpoint combined with cardiovascular death or heart failure hospitalization in patients with chronic, symptomatic HFrEF [10]. Conventionally, clinicians have tended to postpone the commencement of newer heart failure drugs until the outpatient environment because of issues related to hemodynamic instability, alterations in renal function, or hypotension during hospitalization [11]. Nevertheless, recent data also indicate that early administration of guideline-based treatments in hospital treatment can be of valuable benefit [12,13]. The introduction of sacubitril/valsartan when the patients are hospitalized can help to track the treatment tolerance better, guarantee that the patients are exposed to evidence-based treatment earlier on, and possibly help minimize the risks of adverse outcomes in the high-risk post-discharge period [14].

In the setting of HFrEF, barriers such as clinical inertia and lack of proper follow-up might result in a delayed or missed initiation of treatments [15]. Patients should receive optimal guideline-directed pharmacological therapy to reduce all-cause mortality and heart failure readmission in those with HFrEF [16,17]. To adequately reduce the mortality and morbidity associated with chronic heart failure, it is important to treat both mechanisms of disease associated with it, i.e., the increased activation of the renin-angiotensin-aldosterone system (RAAS) and the adrenergic system [18].

The recent literature is starting to investigate the safety and practicability of in-hospital initiation of sacubitril/valsartan; however, there is little real-life data available that compares clinical outcomes in in-hospital versus delayed outpatient initiation [19]. Specifically, the effect of early start on short outcome measures, including rehospitalization, treatment tolerance, and early mortality, is to be assessed further in a variety of patients [20].

This study aims to evaluate whether initiation of sacubitril/valsartan during hospitalization is associated with improved short-term clinical outcomes compared with delayed outpatient initiation among patients hospitalized with HFrEF. Importantly, this study will provide adequate evidence that will guide clinical practice while maximizing the employment of guideline-based therapy. 

## Materials and methods

Study design and data source

This study was a retrospective observational cohort study conducted using data from the Medical Information Mart for Intensive Care (MIMIC)-IV version 3.1 database [21]. The database contains deidentified health-related data associated with patients admitted to intensive care units at a large tertiary academic medical center in the United States. Data were accessed through Google BigQuery following the completion of required credentialing and data use agreements. The database includes detailed information on patient demographics, hospital admissions, diagnoses, medications, and outcomes, which allowed the identification of heart failure hospitalizations and medication exposure.

Study population

The study population included adult patients with hospital admissions associated with heart failure. Hospitalizations were identified using the International Classification of Diseases Ninth Revision (ICD-9) codes beginning with 428 and ICD-10 codes beginning with I50 recorded in the diagnoses table. Among these hospitalizations, patients with documented exposure to sacubitril/valsartan during the index admission or after discharge were included. Each hospitalization was treated as an independent observation, and no clustering adjustment at the patient level was applied, consistent with the study's focus on hospitalization-level outcomes. For hospitalizations with multiple medication records, the earliest recorded administration time was used to define exposure. Hospitalizations with missing data in key exposure or covariate variables were excluded from analyses, resulting in a final analytic cohort of 4,156 hospitalizations.

Variables and measures

The primary exposure variable was the timing of initiation of sacubitril/valsartan, classified as in-hospital initiation when the first recorded administration occurred between admission time and discharge time and delayed outpatient initiation when the first administration occurred after discharge. The primary outcome was 30-day hospital readmission, defined as any subsequent hospital admission within 30 days following discharge from the index hospitalization. The secondary outcome was in-hospital mortality, defined using the hospital expiration flag recorded in the admissions data. Covariates included age at admission, sex, race, and comorbidity burden measured using the Charlson Comorbidity Index. Race was categorized into five groups, including White, Black, Hispanic, Asian, and other or unknown, to ensure adequate sample size within each category.

Missing data

Missing data were minimal for variables included in the analysis. Medication timing variables had less than 0.1% missing values. The exposure variable was missing in 15.9% of the initial dataset, and these observations were excluded prior to analysis. The final analytic cohort included only hospitalizations with complete exposure data. Covariates used in regression models had negligible missingness, and complete case analysis was performed without imputation.

Statistical analysis

Descriptive statistics were used to summarize the baseline characteristics of the study population. Continuous variables were reported as means with standard deviations, and categorical variables were reported as frequencies with percentages. Group comparisons by the exposure variable were conducted using t-tests for continuous variables and chi-squared tests for categorical variables. The association between initiation strategy and 30-day readmission was evaluated using multivariable logistic regression, adjusting for age, sex, race, and the Charlson Comorbidity Index. Results were reported as odds ratios (OR) with 95% confidence intervals (CI). For in-hospital mortality, the presence of zero events in one exposure group resulted in complete separation, and logistic regression could not be performed. Therefore, Fisher's exact test was used to compare mortality between groups. Statistical significance was defined as a two-sided p-value of less than 0.05. All analyses were conducted using Stata Version 18 (StataCorp LLC, College Station, Texas, United States) [22].

Ethical considerations

The study is based on a secondary analysis of deidentified patient data from the MIMIC-IV database. All data in MIMIC-IV are fully deidentified, and access requires completion of a data use agreement and certification in human subjects research. As the study used publicly available deidentified human data, institutional review board approval and individual informed consent were not required.

## Results

Table 1 presents the baseline characteristics of patients according to the timing of sacubitril/valsartan initiation. The study cohort showed a marked imbalance in group sizes, with a relatively small delayed initiation group (n=62) compared to the in-hospital initiation group (n=4,094), which may limit statistical power and should be considered when interpreting the results.

**Table 1:** Baseline characteristics of patients according to timing of sacubitril/valsartan (ARNI) initiation Values are presented as mean and standard deviation for continuous variables and as frequency with column percentage for categorical variables. The analytic sample included 4,156 hospitalizations. Group comparisons were performed using t-tests for continuous variables and chi-squared tests for categorical variables. ARNI: angiotensin receptor-neprilysin inhibitor The table was generated and compiled by the authors using Stata Version 18 (StataCorp LLC, College Station, Texas, United States) [22].

Variable	Delayed initiation (n=62)	In-hospital initiation (n=4,094)	Test statistic	P-value
Age, years, mean (SD)	67.18 (15.80)	67.65 (14.04)	t=-0.26	0.794
Charlson Comorbidity Index, mean (SD)	6.32 (2.57)	6.58 (2.50)	t=-0.79	0.430
Gender, n (%)				
Male	43 (69.35%)	2,267 (55.37%)	χ²=4.84	0.028
Female	19 (30.65%)	1,827 (44.63%)		
Race, n (%)				
White	34 (54.84%)	2,513 (61.38%)	χ²=2.41	0.660
Black	16 (25.81%)	921 (22.50%)		
Hispanic	3 (4.84%)	261 (6.38%)		
Asian	2 (3.23%)	99 (2.42%)		
Other/unknown	7 (11.29%)	300 (7.33%)		
Insurance, n (%)				
Medicaid	5 (8.06%)	567 (13.91%)	χ²=6.41	0.093
Medicare	45 (72.58%)	2,726 (66.86%)		
Other	2 (3.23%)	32 (0.78%)		
Private	10 (16.13%)	752 (18.44%)		

The results indicate that the mean age was similar between groups, with 67.18 (15.80) years in the delayed initiation group and 67.65 (14.04) years in the in-hospital initiation group, with no statistically significant difference (p=0.794). The Charlson Comorbidity Index was also comparable between groups, with 6.32 (2.57) in the delayed group and 6.58 (2.50) in the in-hospital group. A difference was observed in sex distribution, where males accounted for 43 (69.35%) in the delayed group and 2,267 (55.37%) in the in-hospital group and females accounted for 19 (30.65%) and 1,827 (44.63%), respectively, with a statistically significant difference (p=0.028). Race distribution did not differ significantly across groups (p=0.660), with White patients comprising 34 (54.84%) and 2,513 (61.38%), Black patients 16 (25.81%) and 921 (22.50%), Hispanic patients 3 (4.84%) and 261 (6.38%), Asian patients 2 (3.23%) and 99 (2.42%), and other or unknown 7 (11.29%) and 300 (7.33%) in the delayed and in-hospital groups, respectively. Insurance status was similar between groups, with no statistically significant differences observed (p=0.093).

Table 2 presents the multivariable logistic regression analysis for 30-day hospital readmission.

**Table 2:** Multivariable logistic regression analysis of factors associated with 30-day hospital readmission Adjusted OR and 95% CI were estimated using multivariable logistic regression. The model adjusted for age, sex, race, and the Charlson Comorbidity Index. In-hospital initiation and White race were used as reference categories. OR: odds ratio; CI: confidence interval; ARNI: angiotensin receptor-neprilysin inhibitor The table was generated and compiled by the authors using Stata Version 18 (StataCorp LLC, College Station, Texas, United States) [22].

Variable	Adjusted OR	95% CI	P-value
ARNI initiation			
Delayed vs. in-hospital	0.91	0.50-1.67	0.764
Charlson Comorbidity Index	1.04	1.00-1.07	0.035
Age	1.00	0.99-1.00	0.452
Gender			
Female vs. male	0.94	0.80-1.09	0.415
Race			
Black vs. White	1.17	0.98-1.41	0.090
Hispanic vs. White	1.50	1.13-2.01	0.006
Asian vs. White	1.06	0.65-1.72	0.816
Other/unknown vs. White	0.50	0.35-0.72	<0.001

The results indicate that delayed initiation of sacubitril/valsartan was not associated with a difference in 30-day readmission compared with in-hospital initiation (aOR=0.91; 95% CI: 0.50-1.67; p=0.764). The Charlson Comorbidity Index was associated with higher odds of readmission (aOR=1.04; 95% CI: 1.00-1.07; p=0.035). Age and sex were not significantly associated with readmission. Compared with White patients, Hispanic patients had higher odds of readmission (aOR=1.50; 95% CI: 1.13-2.01; p=0.006), while patients categorized as other or unknown had lower odds (aOR=0.50; 95% CI: 0.35-0.72; p<0.001). No significant differences were observed for Black or Asian patients compared with White patients.

Table 3 presents in-hospital mortality according to the timing of sacubitril/valsartan initiation.

**Table 3:** In-hospital mortality by ARNI initiation strategy Values are presented as frequency with percentage. P-value was calculated using Fisher's exact test due to small cell counts and zero events in one group. ARNI: angiotensin receptor-neprilysin inhibitor The table was generated using Stata Version 18 (StataCorp LLC, College Station, Texas, United States) [22].

Variable	Delayed initiation (n=62)	In-hospital initiation (n=4,094)	Fisher's exact p-value
Mortality, n (%)	0 (0%)	54 (1.32%)	1.000

The results indicate that no deaths occurred in the delayed initiation group, while 54 (1.32) deaths were observed in the in-hospital initiation group. There was no statistically significant difference in in-hospital mortality between the two groups.

**Figure 1:**
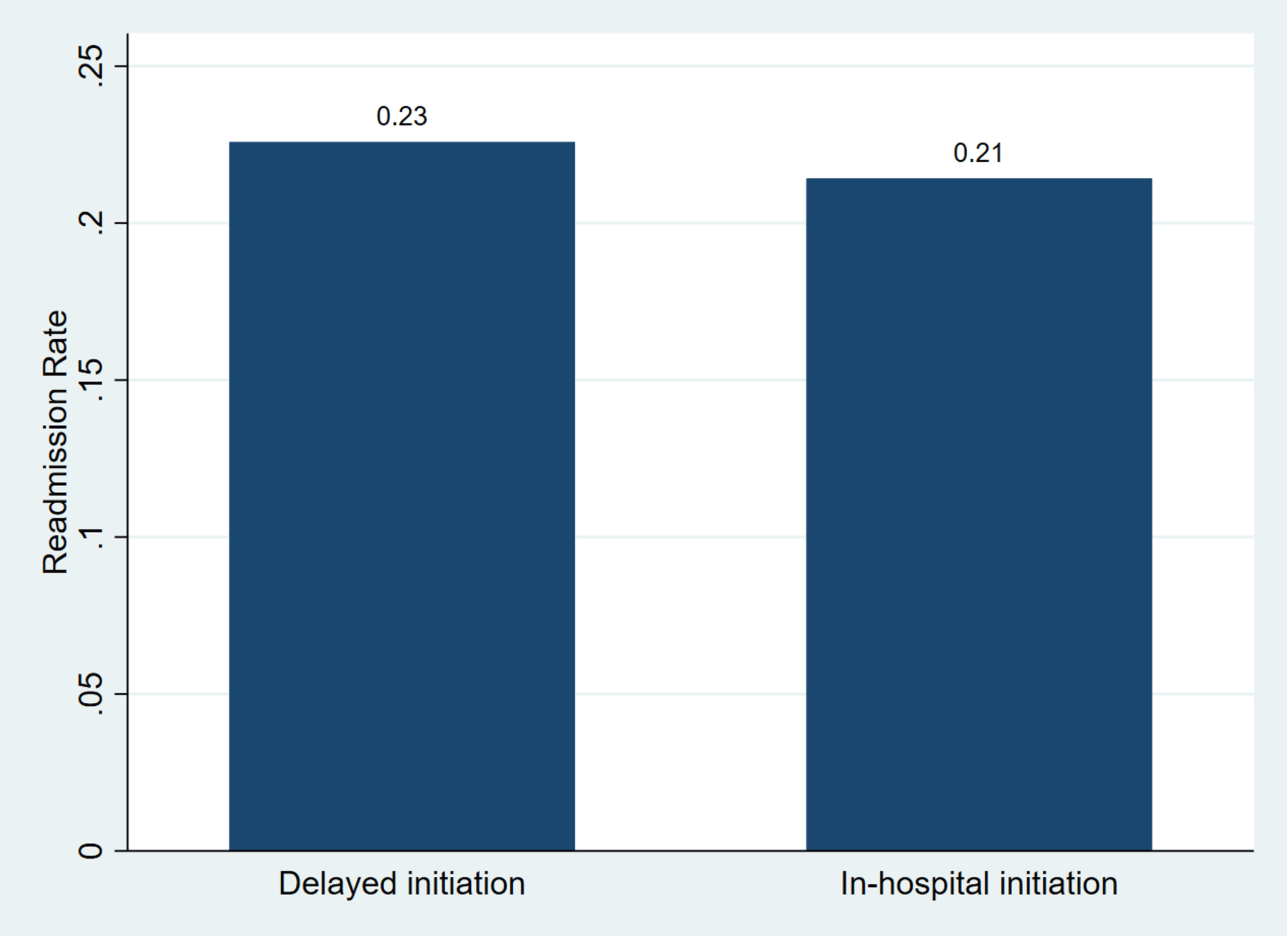
Proportion of 30-day hospital readmission by timing of sacubitril/valsartan initiation Bars represent the proportion of patients with 30-day readmission within each exposure group.

Figure 1 illustrates the proportion of patients with 30-day hospital readmission according to the timing of sacubitril/valsartan initiation.

The results indicate that the proportion of 30-day readmission was 0.23 in the delayed initiation group and 0.21 in the in-hospital initiation group. The difference between groups was small and consistent with the regression analysis in Table 2, showing no statistically significant association between timing of initiation and readmission.

## Discussion

In this study, the timing of sacubitril/valsartan initiation during hospitalization or after discharge was not associated with a difference in 30-day hospital readmission. The adjusted analysis showed similar odds of readmission between delayed and in-hospital initiation groups. Comorbidity burden, measured by the Charlson Comorbidity Index, was associated with higher odds of readmission, while age and sex were not associated with the outcome. Differences were observed across racial groups, with Hispanic patients having higher odds of readmission and patients categorized as other or unknown having lower odds compared with White patients. In-hospital mortality was low and did not differ between groups. These findings align with prior studies of patients with HFrEF, including cohort and clinical trial populations, which show that outcomes are strongly influenced by baseline risk factors such as comorbidity burden rather than a single therapeutic decision point [1,2]. Evidence from clinical trials and observational studies has demonstrated that sacubitril/valsartan improves outcomes in HFrEF, including reductions in recurrent events and improvements in quality of life [6,8]. However, these benefits may depend on sustained use and optimization rather than the exact timing of initiation. Studies evaluating inpatient initiation have shown that early use is feasible and safe, but differences in short-term outcomes may be modest, particularly when follow-up care and treatment adherence are not captured [10,11,14].

Current heart failure management guidelines, including those from the Heart Failure Association of the European Society of Cardiology (ESC) and the American College of Cardiology (ACC), emphasize the importance of early initiation of sacubitril/valsartan as a cornerstone of GDMT for patients with HFrEF [4,13]. These guidelines recommend that sacubitril/valsartan be initiated as soon as patients are hemodynamically stable, ideally during hospitalization or shortly after discharge, to maximize therapeutic benefit and reduce mortality and morbidity [3,9]. The rationale is based on evidence demonstrating that early exposure to ARNI can halt or reverse cardiac remodeling, improve neurohormonal profiles, and thereby improve long-term outcomes [7,18]. Moreover, guideline-directed protocols advocate for the careful monitoring of renal function, blood pressure, and potassium levels during initiation and titration phases to ensure safety and tolerability [9,13]. Guidelines per the American Heart Association suggest classifying heart failure into three classes: heart failure with preserved ejection fraction (≥50%), heart failure with borderline ejection fraction (41-49%), and HFrEF (≤40%) [2]. The advent of sacubitril/valsartan has significantly lowered the risk of cardiovascular death and rehospitalization in patients who identify with the class of HFrEF [10]. Since sacubitril/valsartan has a similar adverse effect profile to angiotensin-converting enzyme inhibitors (ACEIs)/angiotensin receptor blockers (ARBs), monitoring for these effects is essential to ensure drug tolerance. The side effects include renal dysfunction, angioedema, hyperkalemia, dizziness, cough, and hypotension [9]. With this background, it should be noted that similar precautions/contraindications considered for ACEIs/ARBs apply to the use of sacubitril/valsartan. A previous history of angioedema or simultaneous use of ACEIs when initiating sacubitril/valsartan, for instance, is considered a contraindication to the use of the drug [18]. For in-patients started on sacubitril/valsartan, even though the overall risk of drug discontinuation was low, this risk was strongly associated with events of hypotension and renal dysfunction in the form of acute kidney injury, making them strong limiting factors. Outpatients were also noted to show withdrawal from the medication due to renal dysfunction, hypotension, and recovery of the ejection fraction of the left ventricle [19]. Again, medication cost was a strongly implicated reason for limiting drug use especially in the post-discharge period [11,19].

Despite this, real-world practice often reflects hesitancy to start sacubitril/valsartan inpatient due to concerns over hypotension, renal impairment, or other acute factors [11]. Our findings, showing no significant difference in short-term outcomes between in-hospital and delayed outpatient initiation, may partially reflect this cautious approach, underscoring the need for comprehensive care pathways that incorporate guideline recommendations, allow for early but safe initiation, and ensure robust post-discharge follow-up to optimize adherence and dosing [3,5,15]. Future efforts should focus on implementing these guideline-based strategies systematically to overcome clinical inertia and improve patient-centered outcomes.

Several mechanisms may explain the observed findings. Sacubitril/valsartan exerts its effects through neprilysin inhibition and blockade of the renin-angiotensin system, leading to improved hemodynamics and reduced neurohormonal activation [7,18]. These mechanisms may require time and consistent exposure to translate into measurable clinical benefit. Early initiation during hospitalization may allow earlier engagement of these pathways, but the impact on short-term outcomes such as 30-day readmission may be limited if other factors such as volume status, adherence, and follow-up care are not optimized. The association between higher comorbidity burden and increased readmission risk is consistent with the complex clinical profile of patients with heart failure, where multiple coexisting conditions contribute to recurrent hospitalization [1,2]. Differences observed across racial groups should be interpreted with caution, as the study was not designed to assess causal effects and residual confounding may be present. These differences may reflect variations in access to care, social factors, or underlying disease severity described in prior studies of heart failure populations [2].

Strengths and limitations of the study

This study has several strengths and limitations. The use of a large clinical database allowed for the evaluation of real-world patterns of sacubitril/valsartan initiation and short-term outcomes. Key clinical variables such as age, sex, race, and comorbidity burden were included in the analysis.

However, important variables were not available, including ejection fraction measurements, hemodynamic stability at the time of treatment initiation, medication adherence, dose titration, and outpatient follow-up, which may influence outcomes. The exposure variable had missing data for a subset of hospitalizations (15.9%), and complete case analysis was used. This may introduce selection bias if the missingness is not random, potentially affecting the generalizability of the findings.

There was also a marked imbalance in group sizes, with a relatively small delayed initiation group compared to the in-hospital initiation group, which may substantially reduce statistical power and limit comparability between groups. The analysis relied on recorded clinical data rather than self-reported measures, which may limit the capture of patient-level factors such as symptoms and adherence. The study design reflects observational data and does not establish causality.

Future research should incorporate longitudinal follow-up, detailed treatment patterns, and measures of care transitions to better understand how the timing of therapy initiation interacts with broader management strategies.

## Conclusions

This study highlights that the timing of sacubitril/valsartan initiation during hospitalization compared with delayed outpatient initiation was not associated with differences in short-term readmission or in-hospital mortality among patients with HFrEF, although the marked imbalance in group sizes may have limited the statistical power to detect potential differences. Comorbidity burden remained an important factor associated with readmission risk. These findings support the importance of comprehensive management beyond the timing of therapy initiation, including continuity of care and optimization of treatment. From a clinical perspective, early initiation appears feasible but may require integration with structured follow-up to influence outcomes. Future research should focus on longitudinal care patterns, medication titration, and post-discharge management to better define strategies that improve patient outcomes in this population.
